# Metabolic Dysfunction-Associated Steatotic Liver Disease: The Associations between Inflammatory Markers, TLR4, and Cytokines IL-17A/F, and Their Connections to the Degree of Steatosis and the Risk of Fibrosis

**DOI:** 10.3390/biomedicines12092144

**Published:** 2024-09-21

**Authors:** Sorina-Cezara Coste, Olga Hilda Orășan, Angela Cozma, Vasile Negrean, Adela-Viviana Sitar-Tăut, Gabriela Adriana Filip, Adriana Corina Hangan, Roxana Liana Lucaciu, Mihaela Iancu, Lucia Maria Procopciuc

**Affiliations:** 1 4th Department of Internal Medicine, Faculty of Medicine, “Iuliu Hațieganu” University of Medicine and Pharmacy, 400012 Cluj-Napoca, Romania; secara.sorina@umfcluj.ro (S.-C.C.); hilda.orasan@umfcluj.ro (O.H.O.); angelacozma@umfcluj.ro (A.C.); vasile.negrean@umfcluj.ro (V.N.); adela.sitar@umfcluj.ro (A.-V.S.-T.); 2 Department of Anatomy and Embryology, Faculty of Medicine, “Iuliu Hațieganu” University of Medicine and Pharmacy, 400006 Cluj-Napoca, Romania; adrianafilip33@yahoo.com; 3 Department of Inorganic Chemistry, Faculty of Pharmacy, “Iuliu Hațieganu” University of Medicine and Pharmacy, 400012 Cluj-Napoca, Romania; adriana.hangan@umfcluj.ro; 4 Department of Pharmaceutical Biochemistry and Clinical Laboratory, Faculty of Pharmacy, “Iuliu Hațieganu” University of Medicine and Pharmacy, 400012 Cluj-Napoca, Romania; liana.lucaciu@umfcluj.ro; 5 11th Department of Medical Education, Medical Informatics and Biostatistics, “Iuliu Hațieganu” University of Medicine and Pharmacy, 400012 Cluj-Napoca, Romania; 6 Department of Molecular Sciences, “Iuliu Hațieganu” University of Medicine and Pharmacy, 400349 Cluj-Napoca, Romania; lprocopciuc@umfcluj.ro

**Keywords:** MASLD, cytokine IL17, TLR4, inflammatory markers, the PIV, the SII, hepatic steatosis, fibrosis

## Abstract

**Background**: The pathogenesis of MASLD (metabolic dysfunction-associated steatotic liver disease) is driven by environmental, genetic, metabolic, immune, and inflammatory factors. IL-17 and TLR4 determine hepatic steatosis, inflammation, and finally fibrosis. **Objectives**: To explore the associations between the plasma levels of inflammatory markers, TLR4, and the cytokines IL17A/F, as well as their connections with the degree of hepatic steatosis and the risk of hepatic fibrosis (defined by the FIB-4 score) in MASLD patients. **Methods**: The study cohort included 80 patients diagnosed with MASLD. The IL-17A/F and TLR4 serum concentrations were determined using the ELISA method. **Results**: We found a significant difference in the CAR levels (C-reactive protein to albumin ratio) when comparing MASLD patients with severe steatosis to those with mild/moderate steatosis (Student’s t test, t (71) = 2.32, *p* = 0.023). The PIV (pan-immune inflammatory value) was positively correlated with the SII (systemic immune inflammation index), (r = 0.86, *p* < 0.0001) and the CAR (r = 0.41, *p* = 0.033) in MASLD patients with severe steatosis. In contrast, increased values of the LMR (lymphocyte to monocyte ratio) were significantly associated, with decreased levels of the SII (ρ = −0.38, *p* = 0.045). We also found a positive correlation between the CAR and the SII (r = 0.41, *p* = 0.028). In patients with mild/moderate steatosis, a significant positive correlation was observed between the SII and IL17A (r = 0.36, *p* = 0.010), the PIV and the CAR (r = 0.29, *p* = 0.011), the PIV and the SII (r = 0.87, *p* < 0.0001) and the PIV and IL17A (r = 0.3, *p* = 0.036). A negative correlation was observed between the LMR and the SII (r = −0.55, *p* < 0.0001) and the CAR and IL17F (r = −0.37, *p* = 0.011). Regarding the inflammatory markers, the PIV (336.4 vs. 228.63, *p* = 0.0107), and the SII (438.47 vs. 585.39, *p* = 0.0238) had significantly lower levels in patients with an intermediate–high risk of hepatic fibrosis as compared with the patients with a low risk of hepatic fibrosis. The PNI (prognostic nutritional index) (47.16 vs. 42.41, *p* = 0.0392) had significantly different levels in patients with the likelihood of hepatic fibrosis than those with a low risk of hepatic fibrosis. **Conclusions**: Regarding the inflammatory markers, the PIV and the SII hold promise as biomarkers for discriminating between MASLD patients with an intermediate–high risk and those with a low risk of hepatic fibrosis. Our findings underscore the role of IL-17A and its potential relationship with inflammatory markers in MASLD pathogenesis and the progression to hepatic fibrosis.

## 1. Introduction

MASLD stands for metabolic dysfunction-associated steatotic liver disease. It is a recently proposed term that replaces non-alcoholic fatty liver disease (NAFLD) to reflect better the metabolic origins and characteristics of the condition [1]. The transition from NAFLD to MASLD was proposed to capture the metabolic roots of the disease better, moving away from defining it primarily by what it is not (i.e., non-alcoholic) and instead focusing on the underlying metabolic issues. 

MASLD is currently the most prevalent chronic liver disease worldwide, affecting over a third of the global adult population. Its prevalence varies significantly by region, with exceptionally high rates observed in South America (44%) and lower rates in Europe (25%) [2,3]. The global burden of MASLD is predicted to increase, along with the significant healthcare costs associated with managing liver-related complications like fibrosis, cirrhosis, and hepatocellular carcinoma (HCC) in advanced stages [4]. 

MASLD is a spectrum of liver diseases that are characterized by excessive fat accumulation in the liver (steatosis) and associated with metabolic dysfunction, including obesity, insulin resistance, type 2 diabetes, and dyslipidemia [5]. The pathogenesis of MASLD has not been fully elucidated. It is driven by environmental, genetic, metabolic, immune, and inflammatory factors [6]. 

Interleukin-17A (IL-17A) and Interleukin-17F (IL-17F) are pro-inflammatory cytokines that play crucial roles in the immune system, particularly in host defense against bacterial and fungal infections. Both are members of the IL-17 cytokine family, which consists of six members (IL-17A to IL-17F). Among these, IL-17A and IL-17F are the most studied members of the IL-17 family of cytokines and are often co-expressed by Th17 cells, a subset of CD4+ T cells [7]. IL-17 has a significant role in the development and progression of MASLD because it exacerbates inflammation, contributes to fibrosis, and influences metabolic dysfunction [8]. IL-17 signaling increases the production of pro-inflammatory cytokines in the liver, which enhances the recruitment of myeloid cells and T cells, exacerbating inflammation and contributing to the progression of liver disease [9]. Additionally, IL-17 can directly activate hepatic stellate cells (HSCs), crucial in liver fibrosis. This activation leads to increased fibrosis, a characteristic of severe MASLD progression [10].

Toll-like receptor 4 (TLR4) is a critical component of the innate immune system, playing a central role in the body’s first line of defense against microbial infections. TLR4 is part of the larger Toll-like receptor family, which recognizes pathogen-associated molecular patterns (PAMPs) and triggers immune responses. It is primarily activated by ligands such as lipopolysaccharide (LPS) and free fatty acids (FFAs) [11]. TLR4 is expressed on various liver cells, including hepatocytes, Kupffer cells, and hepatic stellate cells. They are elevated in metabolic disorders and play a pivotal role in the progression of MASLD by promoting inflammation, fibrosis, and hepatic steatosis [12]. TLR4 activation triggers inflammatory signaling pathways, notably the MyD88-dependent pathway, which produces pro-inflammatory cytokines like TNF-α and IL-6. The TLR4-MyD88 pathway is closely associated with developing liver fibrosis [13]. TLR4 signaling promotes the activation of hepatic stellate cells, leading to increased extracellular matrix production and fibrosis [14], which creates a tumorigenic environment, promoting the transition from liver damage to cancer. TLR4 activation in HCC enhances tumor growth through multiple signaling pathways, including MAPK, NF-κB, and PI3K/AKT, contributing to cancer cell proliferation and survival. The LIN28A/let-7g miRNA axis is involved in TLR4 regulation, creating a positive feedback loop that promotes HCC growth [15].

It is also involved in lipid metabolism. TLR4 activation by FFAs exacerbates the accumulation of fat in liver cells and enhances the inflammatory environment, promoting fibrogenesis. The continuous activation of TLR4 in this pro-inflammatory setting accelerates the transition from benign fatty liver disease to more severe forms like steatohepatitis, which is strongly associated with HCC [16].

Furthermore, TLR4 deficiency has been shown to reduce hepatic fat content, indicating its critical role in promoting steatosis [17]. TLR4-mediated inflammation also promotes liver regeneration, but chronic activation can lead to dysregulated tissue repair and cancerous transformation. In summary, TLR4 contributes to both the fibrotic and carcinogenic processes in MASLD, playing a central role in the progression from steatosis to cirrhosis and ultimately to HCC [18].

The interaction between IL-17 and TLR4 indicates a synergistic relationship, whereby IL-17 potentiates TLR4-mediated pathways. Together, these molecules contribute to a pro-inflammatory and pro-fibrotic environment, accelerating the progression of MASLD to more severe forms, such as steatohepatitis and cirrhosis [19]. 

It is well-established that chronic inflammation plays a crucial role in developing MASLD [20]. The systemic immune inflammation index (the SII) is a composite index used to evaluate the balance between inflammation and immune response in the body. The SII has gained attention recently as a prognostic marker in various diseases, such as infectious and autoimmune diseases, where a high SII indicates severe inflammation. In contrast, in autoimmune diseases, it may reflect the extent of systemic inflammation [21]. The pan-immune inflammation value (PIV) is a composite biomarker that reflects the overall state of systemic inflammation and immune response. It is derived from routine blood parameters and has been proposed as a prognostic indicator in various clinical settings, particularly in oncology. The PIV can also assess the severity and prognosis of chronic inflammatory diseases [22]. Studies reported excellent performance of other inflammatory markers similar to the PIV and the SII in the diagnosis and prognosis of cancer, cardiovascular disease, and infectious diseases [23].

However, evidence comparing the correlation of inflammatory markers with MASLD is lacking. Also, few clinical studies currently aimed to identify associations between the human cytokines IL17A/F, human TLR4 levels, and MASLD (formerly known as NAFLD). 

Our study aims to examine the connection between the plasma levels of IL-17A/F and TLR4 and the extent of hepatic steatosis, as well as the likelihood of fibrosis in patients with MASLD. Although IL-17 and TLR4 play roles in inflammatory and fibrotic processes in liver disease, their specific functions in MASLD are not fully understood. Understanding how these markers relate to disease severity could help improve the early detection and the risk assessment of liver fibrosis, a major complication of MASLD. However, there are essential questions that need answers: What exactly is the contribution of IL-17 and TLR4 to the development of steatosis and fibrosis in MASLD? Can these markers be reliable indicators of the fibrosis risk in this population? Our study aims to investigate these issues by exploring the connections between IL-17A/F, TLR4, and inflammatory markers, as well as their associations with the severity of steatosis and the likelihood of fibrosis, to identify new biomarkers that could guide future therapeutic strategies.

## 2. Materials and Methods

### 2.1. Study Design and Population

The current study is a prospective, longitudinal cohort study conducted by CF Clinical University Hospital in Cluj-Napoca, Romania, between September 2022 and January 2024. The study was approved by the Ethics Committee of the “Iuliu Hatieganu” University of Medicine and Pharmacy, Cluj-Napoca (approval no. 322/26.07.2018). It was conducted according to the guidelines of the 1975 Declaration of Helsinki. Before enrollment, each patient provided written informed consent. 

The study cohort consisted of 80 patients diagnosed with MASLD, selected based on specific diagnostic criteria. Patients were initially recruited from a larger pool of individuals identified with metabolic syndrome (MS) and non-alcoholic fatty liver disease (NAFLD) through routine clinical assessments. The diagnosis of MS was established if at least three of the following five criteria were present: a. hyperglycemia: fasting glucose level values ≥ 100 mg/dL or medication for elevated glucose; b. low HDL-cholesterol values of <40 mg/dL in men and <50 mg/dL in women or treatment in for low HDL-C; c. hypertriglyceridemia: TG ≥ 150 mg/dL or taking medication for elevated TG; d. waist circumference ≥ 102 cm in men and ≥88 cm in women; e. hypertension, defined as BP ≥ 130/85 mmHg or the presence of antihypertensive treatment.

The inclusion criteria were aligned with the recently updated MASLD diagnostic criteria, which emphasize both liver steatosis and underlying metabolic dysfunction [24,25].

Patients were included if they met the following two primary conditions:

1. Presence of liver steatosis, confirmed via ultrasound imaging.
2. Evidence of metabolic dysfunction, defined by the presence of at least one of five cardiometabolic risk factors:
  - Body mass index (BMI) ≥ 25 kg/m^2^ or waist circumference (WC) > 94 cm (men) or >80 cm (women);
  - Fasting serum glucose ≥ 100 mg/dL, diagnosed type 2 diabetes, or ongoing treatment for diabetes;
  - Blood pressure ≥ 130/85 mmHg or treatment with antihypertensive medication;
  - Plasma triglycerides ≥ 150 mg/dL or use of lipid-lowering medication;
  - Plasma HDL-cholesterol < 40 mg/dL (men) or <50 mg/dL (women) or ongoing lipid-lowering treatment.

To ensure that the sample was representative of the broader MASLD population, we implemented the following steps during patient selection:

1. Stratification by Steatosis Severity: Patients were stratified into three groups based on their degree of hepatic steatosis, mild, moderate, or severe, ensuring that all the stages of disease progression were represented. This stratification allowed us to examine the relationship between metabolic dysfunction and steatosis severity across the MASLD spectrum.
2. The Exclusion of Confounding Conditions: To isolate the effects of metabolic dysfunction on liver health, patients with significant alcohol consumption, other liver diseases (viral hepatitis B and C, Wilson’s disease, autoimmune hepatitis, and hemochromatosis), or drug consumption (amiodarone, methotrexate, glucocorticoids, valproate, and tamoxifen) were excluded. By strictly adhering to these exclusion criteria, we ensured that the sample was more representative of individuals diagnosed specifically with MASLD, rather than those with mixed etiologies.
3. Metabolic Syndrome Criteria as a Foundation: The inclusion of patients initially diagnosed with MS ensured that all the participants presented with a set of metabolic abnormalities that are commonly associated with MASLD.

### 2.2. Data Collection, Measurements, and Venous Blood Sample Collection Preparation

Information related to age, gender, residence status, alcohol consumption, smoking status, and medical history (for comorbidities such as T2D or arterial hypertension) was collected from all the patients included in the study. Their body mass index (BMI) was calculated based on their height and weight using the following formula: weight (kilograms) divided by height (meters) squared. Their waist circumference (WC) was measured at the level of the umbilicus. Their systolic and diastolic blood pressure values were recorded. 

All the study participants fasted for at least eight hours before the blood samples were collected to ensure consistent metabolic and inflammatory marker measurements. Trained medical personnel collected venous blood samples using standard techniques for the usual determinations: complete blood count with platelets; fasting blood glucose (FBG); total cholesterol (TC); HDL-cholesterol (HDL-C); LDL-cholesterol (LDL-C); triglycerides (TG); aminotransferases: alanine aminotransferase (ALT) and aspartate aminotransferase (AST); alkaline phosphatase (AP); total bilirubin (TB); serum albumin (Alb); the international normalized ratio (INR); and C-reactive protein (CRP). The blood samples were immediately transported to the laboratory for analysis. Upon arrival at the laboratory, blood samples in serum separator tubes were centrifuged at 3000 rpm for 10 min to separate the serum from the cellular components. The serum samples were either analyzed immediately or stored at −80 °C until further analysis (ELISA method for the measurement of cytokine levels). The samples in EDTA tubes were analyzed promptly for the complete blood count (CBC), using automated hematology analyzers to ensure accuracy and consistency in cell counts—Hematology Sysmex XN 550 with Sysmex reactive kit (from Sysmex America Inc., Lincolnshire, IL, USA). The biochemical parameters were determined using Biochemistry Integra 400 and COBAS c501 analyzers with a Roche Diagnostics reactive kit (from Roche Hoffman—La Roche Ltd. Basel, Switzerland).

### 2.3. Measurement of Inflammatory Markers

The following inflammatory markers were derived from the blood sample results based on established formulas:

1. Systemic immune inflammation index (SII): (platelet count (10^3^/mmc) × neutrophil count (10^3^/mmc))/lymphocyte count (10^3^/mmc).
2. The pan-immune inflammatory value (PIV): [neutrophil count (10^3^/mmc) × platelet count (10^3^/mmc) × monocyte count (10^3^/mmc)]/lymphocyte count (10^3^/mmc) [26].
3. C-reactive protein to albumin ratio (CAR): CRP (mg/dL)/albumin (g/dL).
4. Lymphocyte to monocyte ratio (LMR): lymphocyte count (10^3^/mmc) / monocyte count (10^3^/mmc).
5. Prognostic nutritional index (PNI): (10 × albumin [g/dL]) + (0.005 × lymphocytes count [10^3^/mmc]) [23].

### 2.4. Measurements of Serum IL17A/F and TLR4 Using the ELISA Method

Serum concentrations of human IL-17A/F and TLR4 were determined using enzyme-linked immunosorbent assay (ELISA) kits (BioVendor Laboratorni medicina a.s.; Elabscience Biotechnology Inc., Houston, TX, USA) according to the manufacturer’s protocols. The specific kits used were as follows: IL-17A (Catalog No: E-EL-H0105), IL-17F (Catalog No: RAF043R), and TLR4 (Catalog No: E-EL-H6123).

The serum samples were added to micro-ELISA plate wells that had been pre-coated with a monoclonal antibody specific to human IL-17A/F and TLR4. After the samples were incubated, a biotinylated detection antibody specific to each marker was added, followed by a Horseradish Peroxidase (HRP) conjugate. The enzyme–substrate reaction was initiated by adding the substrate solution to each well, and after the reaction was stopped, the color turned yellow. Optical density (OD) values were measured using an automated ELISA reader at a wavelength of 450 nm, within 15 min of stopping the reaction. The OD values were directly proportional to the concentrations of IL-17A, IL-17F, and TLR4 in each sample.

Sensitivity and Detection Limits

- IL-17A: The sensitivity of the assay was 18.75 pg/mL, and the limit of detection (LOD) was determined to be 0.5 pg/mL.
- IL-17F: The sensitivity of the assay was 3.3 pg/mL, and the LOD was determined to be 15.5 pg/mL.
- TLR4: The sensitivity of the assay was 18.75 pg/mL, with a minimum detectable concentration of 0.039 ng/mL.

Specificity

The ELISA assays for IL-17A/F and TLR4 demonstrated high specificity. No cross-reactivity or interference was observed between human IL-17A, IL-17F, TLR4, or their analogs, ensuring the reliability and accuracy of the measurements.

### 2.5. Diagnosis and Assessment of Non-Alcoholic Fatty Liver Disease 

The presence of hepatic steatosis was based on the following criteria: a. the diagnosis of hepatic steatosis by imaging methods—abdominal ultrasonography; b. the exclusion of alcohol consumption, defined as >30 g/day for men and >20 g/day for women. 

Steatosis, identified by ultrasonography, was scored using a 0 to 3 scale calculated as follows: absent steatosis (score 0) was defined as normal liver echotexture; mild steatosis (score 1) as a slight and diffuse increase in fine parenchymal echoes with normal visualization of the diaphragm and portal vein borders; moderate steatosis (score 2) as a moderate and diffuse increase in fine echoes with slightly impaired visualization of portal vein borders and diaphragm; and severe steatosis (score 3) as fine echoes with poor or no visualization of portal vein borders, diaphragm, or posterior portion of the right lobe [27].

### 2.6. Definition of Liver Fibrosis 

The Fibrosis-4 index (FIB-4) score was calculated using the following formula: age (years) × AST (U/L)/[PLT(10^9^/L) × √ALT (U/L)]. The results were classified into three risk categories (low, intermediate, and high) according to the two cut-off points for the FIB-4 index, 1.30 and 2.67, as described in the original publications [28].

### 2.7. Statistical Analysis

The demographic, anthropometric, and clinical quantitative variables that followed a normal distribution were summarized using an arithmetic mean and standard deviation, while variables without parametric distributions were described by a median with an interquartile range, IQR = [Q1, Q3], where Q1 = first quartile and Q3 = third quartile. Qualitative clinical features were described by absolute and relative frequency (%). 

The inflammatory parameters (the PIV, the SII, and the CAR) and the cytokines, (IL17A and IL17F) were transformed on a logarithmic scale (base 10) due to their high variability and positive skewness. Their distributions were summarized using geometric mean and geometric standard deviation, while other inflammatory markers (TLR4 and the PNI) did not follow a parametric distribution and were described by a median and an interquartile range (IQR).

The comparisons of inflammatory markers and cytokines between MASLD patients with different degrees of steatosis were performed using ANOVA, Welch’s ANOVA test (in the case of deviations from homogeneity of variances), or the Kruskal–Wallis test. A post-hoc analysis using the Games–Howell test was conducted in the case of statistically significant results for the Welch’s ANOVA test. The significance estimated level obtained from the post-hoc analysis (adjusted-p) was calculated using the Benjamini–Hochberg (BH) method to avoid Type I error inflation.

The comparisons of inflammatory markers and cytokines between MASLD patients with the different risk levels (intermediate–high risk vs. low risk) of hepatic fibrosis, defined based on FIB-4 scores, were performed using the Student’s t test for independent samples. 

The correlation between inflammatory markers and cytokines stratified by the degree of steatosis (mild, moderate, or severe) was tested using Pearson’s coefficient correlations for variables with parametric distributions or Spearman’s coefficient for variables with deviations from parametric distributions. 

All the statistical analyses were performed in R software, version 4.4.0. The results of the two-tailed statistical tests were considered statistically significant if the estimated significance level of ***p*** was lower than the level of significance, set at 0.05. 

## 3. Results

### 3.1. Description of the Studied MASLD Sample

The current study included 80 patients diagnosed with MASLD, divided into three groups, based on their degree of hepatic steatosis. The MASLD patients’ mean age was 61.8 (11.0) years, with a similar sex distribution across the three studied groups (Table 1). Our analysis identified significant differences in the means of the BMI across the groups (Welch’s ANOVA test, F [2.0, 50.7] = 7.79, *p* = 0.001). The post-hoc analysis revealed significant differences between the patients with severe steatosis and the patients with moderate steatosis (Games–Howell test, adjusted-p = 0.005) and between the patients with severe steatosis and those with mild steatosis (Games–Howell test, adjusted-p = 0.001). We also found significant differences in the mean values of waist circumference (Welch’s ANOVA test, F [2.0, 50.5] = 11.64, *p* < 0.001), with patients with severe steatosis having a higher abdominal circumference, in mean, than those with moderate or mild steatosis (mean, 95% CI: 121.6 [115.6, 128.0] vs. 101.4 [95.4, 107.0] vs. 100.8 [94.1, 108.0]).

Conversely, no significant differences were found in arterial hypertension and obesity (related to the abdominal circumference). Still, the frequency of diabetes mellitus was significantly different between the studied groups (*p* = 0.021), with a higher frequency observed in patients with severe steatosis (55.2% vs. 39.3% vs. 17.4%).

### 3.2. Associations of Inflammatory Markers, IL17 Cytokines, TLR4, and Degree of Steatosis

Table 2 shows the relationship between inflammatory markers and the degree of steatosis among the MASLD sample. The results indicated no significant associations between all the inflammatory markers and the degree of steatosis, except for the association between the CAR and degree of steatosis, which showed a trend toward statistical significance (*p* = 0.08). The association was statistically significant when comparing MASLD patients with severe steatosis to those with mild/moderate steatosis (Student’s *t* test, t (71) = 2.32, *p* = 0.023).

### 3.3. Correlations between Inflammatory Markers, Cytokines, and TLR4, Stratified by Degree of Steatosis (Severe Steatosis vs. Mild/Moderate Steatosis)

The PIV was positively correlated with the SII (r = 0.86, *p* < 0.0001) and the CAR (r = 0.41, *p* = 0.033) in MASLD patients with severe steatosis. At the same time, increased values of the LMR were significantly associated with decreased levels of the SII (ρ = −0.38, *p* = 0.045). We also found a positive correlation between the CAR and the SII (r = 0.41, *p* = 0.028). In the same group, we noticed a positive correlation between the SII and IL17F, with a tendency toward statistical significance (r = 0.35, *p* = 0.064). No other cytokines were significantly associated with inflammatory markers in the severe steatosis group (Table 3).

However, in patients with mild/moderate steatosis, a significant positive correlation was observed between the SII and IL17A (r = 0.36, *p* = 0.010), the PIV and the CAR (r = 0.29, *p* = 0.011), the PIV and the SII (r = 0.87, *p* < 0.0001), and the PIV and IL17A (r = 0.3, *p* = 0.036). Significant negative correlations were observed between the LMR and the SII (r = −0.55, *p* < 0.0001) and the CAR and IL17F (r = −0.37, *p* = 0.011).

### 3.4. Associations between Inflammatory Markers, IL17 Cytokines, TLR4 and a Positive FIB-4 Score for Hepatic Fibrosis in MASLD Patients

**Figure 1:**
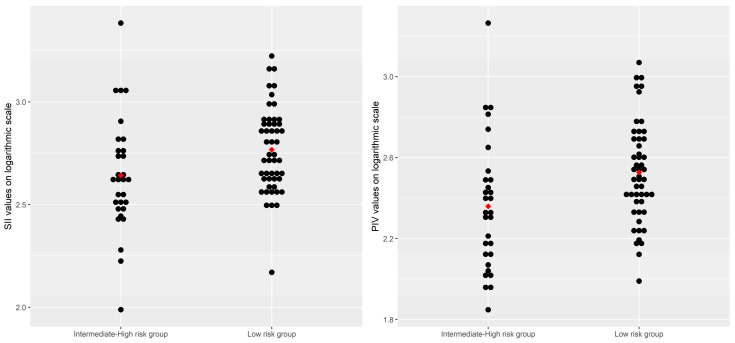
Distributions of the SII and the PIV values on the logarithmic scale by risk levels of hepatic fibrosis, based on FIB-4 scores. Note: each point on the plot represents an individual value of the parameter of interest, transformed on the logarithmic scale; the points in red represent the arithmetic mean values of the inflammatory parameters.

As shown in Table 4, the inflammatory markers, including the PIV (Gmean = 336.40, 95% CI: [286.25, 395.34] vs. Gmean = 228.63, 95% CI: [173.19, 301.81], *p* = 0.0107) and the SII (Gmean = 438.47, 95% CI: [345.36, 556.67] vs. Gmean = 585.39, 95% CI: [511.28, 670.23], *p* = 0.0238), had significantly lower levels in patients with a low risk of hepatic fibrosis than in patients with an intermediate–high risk of hepatic fibrosis. Conversely, the PIN (median, IQR: 47.16 [41.21, 48.76] vs. 42.41 [40.02, 46.21], *p* = 0.0392) had significantly higher levels in patients with an intermediate–high risk of hepatic fibrosis than in patients with a lower risk of hepatic fibrosis. Although the CAR values were, on average, higher in MASLD patients with an intermediate–high risk of hepatic fibrosis, the observed difference had only a trend toward statistical significance (Table 4, Figure 1).

## 4. Discussion

The shift from NAFLD to MASLD reflects the need to better understand the role of metabolic dysfunction and the immune and inflammatory pathophysiological mechanisms underlying the disease. Clinical research is essential to elucidate these mechanisms, identify biomarkers for early detection, and develop targeted therapies [5].

This study investigated the possible associations between the plasma levels of inflammatory markers, IL17A/F, and TLR4 and their connections with the degree of steatosis and hepatic fibrosis among patients with MASLD. This is the first study in Romania to explore the inflammatory process in MASLD by analyzing the role of IL17A/F, TLR4, and potential inflammatory markers. Due to its continuously increasing incidence and prevalence, this condition is a real public health problem nationally and globally.

In our study, 80 patients diagnosed with MASLD were divided into three groups, based on their degree of hepatic steatosis: mild, moderate, or severe steatosis. Our research indicates that among patients with MASLD, there are significant differences in several key variables, depending on the degree of hepatic steatosis. Patients with severe steatosis have a higher BMI, a larger waist abdominal circumference, and a higher frequency of diabetes mellitus compared to those with mild or moderate steatosis, consistent with the association between obesity and metabolic risk factors. There are no significant differences in other cardiometabolic features, like arterial hypertension or lipid levels, across the groups.

Our study aligns with the findings of Garcia et al. (2021), which demonstrated that serum IL-17 levels were significantly higher in individuals with obesity (34.99 pg/mL), with positive correlations between IL-17 levels and both the BMI and waist circumference (OR of 0.413 and 0.337, respectively, both significant at *p* < 0.001) [29].

Similar to Garcia et al., we found that patients with severe steatosis—often associated with a higher BMI and a larger waist circumference—also exhibited elevated IL-17 levels. This reinforces the role of IL-17 as a biomarker that is linked to obesity and metabolic risks. Additionally, the positive correlations identified between IL-17 and other metabolic markers, such as triglycerides and insulin levels, support the idea that IL-17 is a key player in the metabolic dysfunction associated with MASLD [29]. While Sumarac-Dumanovic et al. (2009) did not observe significant correlations between IL-17 and anthropometric indices like the BMI or waist circumference in their population, the discrepancy with our findings, as well as those of Garcia et al. (2021), may be attributed to variations in study design, population characteristics, or sample sizes [29,30]. Overall, our results, in conjunction with the conclusions of these studies, further suggest that IL-17 is an important predictive marker for the metabolic complications linked to obesity and MASLD. This is also consistent with Esposito et al. (2003), who highlighted the systemic inflammatory response associated with obesity, mirroring the inflammatory patterns observed in our MASLD cohort [31].

Our study’s findings regarding inflammatory markers and hepatic fibrosis are consistent with those of Jiang et al. (2024), which identified significant associations between the pan-immune inflammatory value (PIV) and the likelihood of hepatic fibrosis in MASLD patients [26]. Specifically, we found that the PIV, the PNI, and the SII were significantly associated with fibrosis likelihood, as defined by the FIB-4 score (*p* = 0.0107, *p* = 0.0392, respectively *p* = 0.0238), reinforcing the importance of systemic inflammatory markers in assessing the fibrosis risk. These results, alongside those from Jiang et al., emphasize the relevance of the PIV and the SII as reliable indicators of fibrosis in MASLD, even in the absence of significant changes in cytokine levels [26].

Furthermore, our stratification of patients by steatosis severity revealed positive correlations between the PIV and both the SII and the CAR in those with severe steatosis, while significant associations between IL-17A and markers like the SII and the PIV were observed in patients with mild/moderate steatosis. These observations echo findings from Demiroz et al. (2022), who also demonstrated the role of inflammatory markers like the PIV in relation to the severity of hepatosteatosis [32]. However, while Demiroz et al. reported no significant association for the SII, our study found that the SII plays a critical role in fibrosis progression in MASLD, especially when correlated with other markers such as the CAR and IL-17A [32].

On the other hand, our study’s lack of a statistically significant correlation between TLR4 levels and the FIB-4 index contrasts with the results of Cengiz et al. (2015), who reported a strong relationship between TLR4 and liver fibrosis stages. Their study found a positive correlation between serum TLR4 levels and liver fibrosis severity, with levels increasing as the fibrosis stage progressed (area under the curve (AUC) values of 0.862 for mild fibrosis (≥F1), 0.810 for significant fibrosis (≥F2), and 0.905 for advanced fibrosis (≥F3), all statistically significant (*p* < 0.001). TLR4 also exhibited a superior predictive capacity for identifying significant and advanced fibrosis stages, outperforming other non-invasive fibrosis scores like APRI and FIB-4 [33]. This discrepancy may be due to differences in the patient populations, methodologies, or disease characteristics, highlighting the complexity of TLR4’s role in MASLD [33]. Nonetheless, we observed that TLR4 levels were elevated in patients with a higher likelihood of fibrosis, indicating that while the correlation was not statistically significant, TLR4 still plays a role in the disease’s inflammatory and fibrotic processes, consistent with previous clinical findings.

Furthermore, the study performed by Vespasiani-Gentilucci et al. (2015) investigated the role of hepatic TLR4 in MAFLD, focusing on its association with portal inflammation and fibrosis. The authors found that TLR4 expression was particularly elevated in hepatic progenitor cells, biliary cells, and portal/septal macrophages. The study also demonstrated that the serum levels of lipopolysaccharide-binding protein (LBP), a marker for lipopolysaccharide (LPS) activity, were higher in MAFLD patients and correlated with the severity of fibrosis and inflammation. This suggests that TLR4, through LPS-induced signaling, plays a pivotal role in driving the inflammatory and fibrogenic processes in NAFLD. The findings underscore the importance of TLR4 as a potential therapeutic target for mitigating liver inflammation and fibrosis in MAFLD patients [34].

The findings of our study, combined with those previously mentioned in other research, support the important role of systemic inflammatory markers, such as the PIV, the SII, and also the role of TLR4 in the progression of MASLD.

Our study acknowledges certain limitations. The single-center design may limit the external validity, potentially affecting the generalizability of the results to MASLD patients in different regions or healthcare settings. Additionally, as a post-pandemic study with only 80 patients, the sample size, while adequate for a preliminary analysis, may limit the generalizability of the findings and the statistical power to detect smaller effects or interactions. The initial cohort was selected based on NAFLD and metabolic syndrome (MS) criteria, but the final population was refined to include only those meeting the more specific MASLD criteria, reflecting a crucial shift in focus from steatosis alone to the broader metabolic dysfunction now recognized as the primary driver of liver disease. Another potential limitation of this study is the absence of detailed data or control over additional comorbidities and medication use, which may affect inflammatory markers levels.

While our study has limitations, it contributes valuable regional data on MASLD in Romania and offers insights from an under-represented Eastern European population. Our comprehensive approach examined multiple inflammatory markers and cytokine levels, providing new insights into MASLD progression and potential biomarkers for early detection. We also identified correlations between inflammatory markers and different stages of the disease, demonstrating their association with the hepatic fibrosis risk. Additionally, our study highlights the relevance of novel biomarkers like IL-17A/F and TLR4, suggesting their potential for further research into therapeutic interventions for MASLD.

## 5. Conclusions

Inflammatory markers such as the PIV and the SII could be markers for the pathogenesis of hepatic fibrosis. Our results underscore the potential utility of the PIV and the SII as biomarkers for assessing disease severity in MASLD. An interaction between IL17A and these inflammatory markers played a role in the severity of MASLD.

## Figures and Tables

**Table 1:** Demographic, anthropometric, and clinical characteristics of MASLD patients.

Characteristics	Overall (MASLD) (n = 80)	Mild Steatosis Group (n_1_ = 23)	Moderate Steatosis Group (n_2_ = 28)	Severe Steatosis Group (n_3_ = 29)	*p* ^(a)^
Demographics					
Age (years)	61.8 (11.0)	61.9 (10.4)	60.9 (11.4)	62.7 (11.4)	0.829
Sex, n (%)					0.541
Female	57 (71.3)	16 (69.6)	22 (78.6)	19 (65.5)	
Male	23 (28.8)	7 (30.4)	6 (21.4)	10 (34.5)	
Place of residence, n (%)					0.204
Rural	50 (62.5)	11 (47.8)	20 (71.4)	19 (65.5)	
Urban	30 (37.5)	12 (52.2)	8 (28.6)	10 (34.5)	
Anthropometrics					
BMI (kg/m^2^)	33.4 (5.8)	31.0 (4.1)	31.9 (4.5)	36.8 (6.7)	0.001 *
Waist circumference (cm)	108.6 (18.7)	100.8 (12.5)	101.4 (13.4)	121.6 (20.5)	0.0001 *
Cardiometabolic features					
SBP (mmHg)	141.4 (17.7)	140.7 (15.9)	138.2 (15.3)	145.1 (20.7)	0.326
DBP (mmHg)	86.1 (10.9)	87.1 (11.9)	84.6 (10.8)	86.8 (10.4)	0.672
Fasting blood glucose (mg/dL)	99 (89 to 117)	98 (91 to 103)	102 (84 to 117)	100 (92 to 132)	0.322
HDL-cholesterol (mg/dL) ^(a)^	49.1 (11.5)	52.5 (10.6)	49.1 (11.1)	46.0 (12.3)	0.136
LDL-cholesterol (mg/dL)	114.7 (46.7)	114.6 (41.1)	121.2 (41.2)	108.4 (55.6)	0.590
Total cholesterol (mg/dL)	195.2 (52.2)	200.6 (49.8)	203.4 (50.5)	182.9 (55.1)	0.286
Triglycerides (mg/dL)	138 (102 to 191)	109 (101 to 196)	152 (107 to 193)	141 (114 to 179)	0.709
ALT (mg/dL)	18 (14 to 28)	19 (17 to 24)	17 (14 to 23)	18 (14 to 33)	0.622
AST (mg/dL)	20 (17 to 24)	20 (18 to 23)	20 (17 to 24)	19 (16 to 26)	0.950
AST/ALT ratio	1.1 (0.4)	1.1 (0.3)	1.1 (0.3)	1.1 (0.6)	0.919
Alkaline phosphatase (U/I) ^(b)^	82.4 (25.3)	77.2 (26.4)	84.8 (19.8)	84.2 (29.0)	0.515
GGT (U/I) ^(c)^	25 (20 to 38)	26 (18 to 37)	24 (17 to 33)	31 (22 to 42)	0.092
Total bilirubin (mg/dL)	0.5 (0.3 to 0.7)	0.5 (0.3 to 0.8)	0.4 (0.3 to 0.6)	0.5 (0.3 to 0.7)	0.263
Albumin (g/dL)	4.4 (0.5)	4.6 (0.6)	4.4 (0.5)	4.3 (0.4)	0.093
Hgb (g/dL)	14.0 (1.6)	13.9 (1.3)	14.1 (1.8)	14.1 (1.6)	0.843
Hct (%)	42.1 (4.1)	41.2 (3.2)	42.3 (4.4)	42.6 (4.4)	0.465
Hypertension, n (%)	68 (85.0)	19 (82.6)	22 (78.6)	27 (93.1)	0.328
T2DM, n (%)	31 (38.8)	4 (17.4)	11 (39.3)	16 (55.2)	0.021 *
Obesity, n (%)	68 (85.0)	19 (82.6)	23 (82.1)	26 (89.7)	0.731

ALT—alanine amino transferase; AST—aspartate amino transferase. Data were described using mean (standard deviation), n = absolute frequency an estimate from the Welch’s ANOVA test, ANOVA test, Kruskal–Wallis test, Chi-squared test, and Fisher’s exact test; * significant result: *p* < 0.05. ^(a)^ Complete case data: n_1_ = 23, n_2_ = 28; n_3_ = 28; ^(b)^ complete case data: n_1_ = 23, n_2_ = 27; n_3_ = 29; ^(c)^ complete case data: n_1_ = 22, n_2_ = 26; n_3_ = 29.

**Table 2:** Distributions of inflammatory markers, IL17 cytokines, and TLR4 in MASLD patients with mild, moderate, and severe steatosis.

Characteristics	Mild Steatosis Group (n_1_ = 23)	Moderate Steatosis Group (n_2_ = 28)	Severe Steatosis Group (n_3_ = 29)	*p* ^(a)^
PLT (10^3^/uL)	237.7(55.1)	256.6 (49.5)	249.6 (71.5)	0.531
WBC ^(a)^ (10^3^/uL)	7.3 (6.3 to 8.3)	8.3 (6.8 to 8.9)	8.5 (6.9 to 9.6)	0.186
Neutrophils (10^3^/uL)	4.5 (1.5)	5.1 (1.9)	5.1 (1.5)	0.368
Lymphocytes (10^3^/uL)	2.1 (1.9 to 2.4)	2.0 (1.6 to 2.7)	2.2 (1.9 to 2.7)	0.673
Monocytes (10^3^/uL)	0.5 (0.1)	0.6 (0.2)	0.6 (0.2)	0.226
PCR ^(b)^ (mg/dL)	0.3 (0.1 to 0.5)	0.3 (0.2 to 0.6)	0.5 (0.2 to 0.9)	0.184
ESR ^(c)^ (mm/1h)	10.0 (4.0 to 13.0)	10 (4.0 to 17.3)	19 (8.0 to 26.0)	0.157
PIV	236.4 (1.7)	323.1 (2.0)	310.3 (2.0)	0.199
LMR	4.5 (1.4)	4.0 (1.7)	4.1 (1.6)	0.561
CAR ^(d)^	0.05 (2.93)	0.07 (2.41)	0.11 (3.51)	0.084
PNI	43.9 (42.5 to 48.4)	43.9 (40.1 to 47.8)	41.8 (40.0 to 46.2)	0.196
SII	462.3 (1.6)	577.7 (1.7)	530.2 (1.9)	0.367
IL17A	109.9 (3.5)	89.9 (2.1)	124.3 (2.5)	0.455
IL17F	12.6 (1.5)	12.9 (1.4)	12.6 (1.3)	0.972
TLR4	904.5 (614.5 to 1014.1)	711.5 (572.9 to 887.1)	779.1 (636.6 to 926.9)	0.484

PIV—pan-immune inflammatory value; LMR—lymphocyte to monocyte ratio; CAR—C-reactive protein to albumin ratio; PNI—prognostic nutritional index; SII—systemic immune inflammation index. Data were described by arithmetic mean (standard deviation) or geometric mean (Gmean) and geometric standard deviation for the variables PIV, CAR, SII, IL17A, and IL17F, which were transformed on the logarithmic scale or the median (IQR); ^(a)^ complete case data: n_1_ = 23, n_2_ = 27, n_3_ = 29; ^(b)^ complete case data: n_1_ = 20, n_2_ = 25, n_3_ = 28; ^(c)^ complete case data: n_1_ = 21, n_2_ = 26, n_3_ = 29; ^(d)^ complete case data: n_1_ = 20, n_2_ = 25, n_3_ = 28.

**Table 3:** Matrix of correlations between inflammatory markers, IL17 cytokines, and TLR4 in MASLD patients stratified by steatosis degree.

Groups /Variables	the PIV	the LMR	the CAR	the PNI	the SII	IL17A	IL17F	TLR4
**Severe steatosis Group**								
the PIV	1.00							
the LMR	0.13 (0.518)	1.00						
the CAR	0.41 (0.033 *)	−0.01 (0.958)	1.00					
the PNI	0.12 (0.549)	0.13 (0.518)	−0.03 (0.897)	1.00				
the SII	0.86 (<0.0001 *)	−0.38 (0.045 *)	0.41 (0.028 *)	0.03 (0.869)	1.00			
IL17A	0.09 (0.640)	−0.30 (0.11)	−0.08 (0.617)	−0.12 (0.539)	−0.02 (0.912)	1.00		
IL17F	0.35 (0.061)	−0.11 (0.556)	0.10 (0.704)	−0.04 (0.851)	0.35 (0.064)	0.09 (0.639)	1.00	
TLR4	−0.16 (0.402)	−0.05 (0.795)	−0.11 (0.586)	−0.16 (0.401)	−0.09 (0.624)	0.18 (0.357)	0.11 (0.573)	1.00
**Mild/moderate steatosis Group**								
	**the PIV**	**the LMR**	**the CAR**	**the PNI**	**the SII**	**IL17A**	**IL17F**	**TLR4**
the PIV	1.00							
the LMR	−0.71 (<0.001 *)	1.00						
the CAR	0.29 (0.011 *)	0.11 (0.472)	1.00					
the PNI	−0.05 (0.729)	−0.15 (0.305)	−0.15 (0.324)	1.00				
the SII	0.87 (<0.0001 *)	−0.55 (<0.0001 *)	0.27 (0.078)	−0.12 (0.403)	1.00			
IL17A	0.30 (0.036 *)	−0.15 (0.283)	−0.11 (0.481)	0.16 (0.267)	0.36 (0.010 *)	1.00		
IL17F	−0.11 (0.435)	−0.09 (0.542)	−0.37 (0.011 *)	0.26 (0.068)	−0.18 (0.198)	−0.06 (0.687)	1.00	
TLR4	0.07 (0.649)	0.16 (0.253)	0.15 (0.322)	−0.16 (0.257)	−0.05 (0.722)	0.21 (0.138)	−0.20 (0.165)	1.00

PIV—pan-immune inflammatory value; LMR—lymphocyte to monocyte ratio; CAR—C-reactive protein to albumin ratio; PNI—prognostic nutritional index; SII—systemic immune inflammation index. Pearson’s coefficient correlation was used for the variables PIV, CAR, SII, IL17A, and IL17F, transformed on the logarithmic scale. Spearman’s coefficient correlation was used for the variables with deviations from Gaussian distribution; * significant results: *p* < 0.05.

**Table 4:** Distributions of inflammatory markers and cytokines, TLR4 levels based on a cut-off of FIB-4 score.

Variables	Patients with Low Risk for Significant Hepatic Fibrosis (FIB-4 Score < 1.30) (n = 50)	Patients with Intermediate–High Risk for Significant Hepatic Fibrosis (FIB-4 Score ≥ 1.30) (n = 30)	*p* ^(a)^
PIV	336.40 (1.76)	228.63 (2.10)	0.0107 *
LMR	4.17 (1.44)	4.19 (1.72)	0.2565
CAR	0.10 (2.65)	0.06 (3.54)	0.0626
PNI	42.41 (40.02 to 46.21)	47.16 (41.21 to 48.76)	0.0392 *
SII	585.39 (1.61)	438.47 (1.90)	0.0238 *
IL17A	103.80 (2.17)	112.80 (3.50)	0.7447
IL17F	12.60 (1.42)	12.89 (1.39)	0.7707
TLR4	809.01 (585.95 to 997.93)	715.76 (596.12 to 818.77)	0.1538

PIV—pan-immune inflammatory value; LMR—lymphocyte to monocyte ratio; CAR—C-reactive protein to albumin ratio; PNI—prognostic nutritional index; SII—systemic immune inflammation index. Data were described using geometric mean (Gmean) and geometric standard deviation for the variables PIV, CAR, SII, IL17A, and IL17F, which were transformed on the logarithmic scale, while variables with no parametric distributions were described using median (IQR); ^(a)^ estimated from Student-t test or Mann-Whitney test; * significant results: *p* < 0.05.

## Data Availability

The original contributions presented in the study are included in the current article, and further inquiries can be directed to the first author.
